# Physical activity is prospectively associated with spinal pain in children (CHAMPS Study-DK)

**DOI:** 10.1038/s41598-017-11762-4

**Published:** 2017-09-14

**Authors:** Claudia Franz, Niels Christian Møller, Lars Korsholm, Eva Jespersen, Jeffrey J. Hebert, Niels Wedderkopp

**Affiliations:** 1 Spine Centre of Southern Denmark, Hospital Lillebaelt, Middelfart, Institute of Regional Health Services Research, University of Southern Denmark, Odense, Denmark; 2 Center of Research in Childhood Health, Department of Sports Science and Clinical Biomechanics, University of Southern Denmark, Odense, Denmark; 3 Independent Statistical Consultant, Copenhagen, Denmark; 4 Department of Rehabilitation, Odense University Hospital, Institute of Clinical Research, University of Southern Denmark, Odense, Denmark; 5 School of Psychology and Exercise Science, Murdoch University, Murdoch, Australia; 6 Faculty of Kinesiology, University of New Brunswick, Fredericton, Canada; 7 The Sports Medicine Clinic, Orthopaedic Department, Hospital of Lillebaelt and The Institute of Regional Health Service Research, University of Southern Denmark, Odense, Denmark

## Abstract

Spinal pain and physical inactivity are critical public health issues. We investigated the prospective associations of physical activity intensity with spinal pain in children. Physical activity was quantified with accelerometry in a cohort of primary school students. Over 19 months, parents of primary school students reported children’s spinal pain status each week via text-messaging (self-reported spinal pain). Spinal pain reports were followed-up by trained clinicians who diagnosed each child’s complaint and classified the pain as non-traumatic or traumatic. Associations were examined with logistic regression modeling using robust standard errors and reported with odds ratios (OR). Children (n = 1205, 53.0% female) with mean ± SD age of 9.4 ± 1.4 years, participated in 75,180 weeks of the study. Nearly one-third (31%) of children reported spinal pain, and 14% were diagnosed with a spinal problem. Moderate intensity physical activity was protectively associated with self-reported [OR(95%CI) = 0.84(0.74, 0.95)], diagnosed [OR(95%CI) = 0.79(0.67, 0.94)] and traumatic [OR(95%CI) = 0.77(0.61, 0.96)] spinal pain. Vigorous intensity physical activity was associated with increased self-reported [OR(95%CI) = 1.13(1.00, 1.27)], diagnosed [OR(95%CI) = 1.25(1.07, 1.45)] and traumatic [OR(95%CI) = 1.28(1.05, 1.57)] spinal pain. The inclusion of age and sex covariates weakened these associations. Physical activity intensity may be a key consideration in the relationship between physical activity behavior and spinal pain in children.

## Introduction

Spinal pain and physical inactivity represent two critical public health issues. Spinal pain is the leading cause of disability worldwide^1^. Annual estimates of spinal pain incidence range from 12% to 33%^2^, with prevalence estimates in young people ranging from 5%^3^ to 86%^4^. The wide variability of these estimates may result from measurement differences, such as the use of self- or parent-reported outcomes, pain definitions, and recall bias^5, 6^.

Physical activity is essential to normal development^7^ and health^8^ in youth. Previous studies have reported physical activity behavior to be related to back pain in young people^9, 10^. One study found increased physical activity to be associated with back pain^11^, another reported no association^12^, and a third had mixed findings^5^. These studies relied on self-reported estimates of physical activity volume and intensity. Self-reported physical activity instruments are subject to bias resulting from errors of recall, exaggerated perceptions of time and effort, and social desirability^13, 14^. Accordingly, self-reported physical activity is often overestimated^14^. Accelerometry provides an objective measure of physical activity behavior^13^, including the ability to quantify the intensity of activity.

Previous longitudinal studies exploring the associations between objectively measured physical activity behavior and spinal pain have reported conflicting findings. Two studies found no association between objectively measured physical activity levels and self-reported spinal pain^15, 16^, while one study suggested that high levels of physical activity protect against future spinal pain^17^. However, limitations of the measures of physical activity and pain may be important sources of bias in these studies. To our knowledge, no prior studies have included objectively measured physical activity and tracking of clinician-assessed spinal pain in children. Therefore, the true nature of the relationship between physical activity exposures and spinal pain outcomes remains unclear.

The aim of this study was to identify the associations of physical activity exposures with future spinal pain occurrences in children. Specifically, we examined for prospective associations between overall physical activity and time spent in different intensities of physical activity with self-reported and clinically diagnosed spinal pain.

## Methods

### Study design

We undertook a prospective cohort study nested in the Childhood Health, Activity, and Motor Performance School Study Denmark (CHAMPS Study–DK). The CHAMPS Study-DK is a quasi-experimental trial designed to investigate the effects of physical education on the health, physical activity behavior, and motor performance of primary school students^18^.

All 19 public primary schools in the municipality of Svendborg, Denmark, were invited to participate in the study. Ten schools participated, with students in six schools receiving additional physical education lessons (270 minutes per week) and students in four schools receiving the traditional quantity of physical education (90 minutes per week). The study has been described in detail previously^19^.

Physical activity exposures were measured during two periods (November to January 2009/2010 and August to October 2010) of seven contiguous days. Spinal pain outcomes were measured each week from November 2009 to June 2011.

### Study participants

Study participants comprised primary school students enrolled in preschool (mean [SD] age = 6.9 [0.4] years) through sixth (mean [SD] age = 12.4 [0.5] years) grade. The Regional Scientific Ethics Committee for Southern Denmark approved the project (ID S20080047), which was registered with the Danish Data Protection Agency (J.nr. 2008-41-2240). The study was performed in accordance with relevant guidelines and regulations. Written informed consent was obtained from parents, and the child gave verbal acceptance prior to enrollment and before each clinical examination.

### Physical activity exposures

Physical activity was objectively measured with GT3X Actigraph accelerometers (Actigraph, Pensacola Florida) during two seven-day measurement phases. Physical activity exposures were classified in four ways. Mean counts per minute (CPM) were used as a measure of average physical activity intensity. We also applied cut points to identify time (proportion of the day) in sedentary, light, moderate, and vigorous physical activity intensities^20, 21^.

Trained research staff fit each child with a customized elastic belt that secured the accelerometer to the child’s right hip^22^. The children were instructed to wear the device from the time they awoke until they went to bed for seven consecutive days, and to remove the device only when bathing or swimming.

A customized software program was used to process the accelerometry data (Propero, version 1.0.18, University of Southern Denmark, Odense, Denmark). The accelerometers recorded physical activity data every 2 seconds and these data were subsequently collapsed into 10-second epochs^23^. The signals were digitized and passed through a filter with band limits of 0.25–2.5 HZ to help eliminate extraneous accelerations not resulting from human movement (e.g., vibration).

All data were visually inspected, and fixed time points were applied to define a standard day^24^. Absences of accelerations lasting 30 consecutive minutes or more were interpreted as ‘accelerometer not worn’. Physical activity data were included in the analyses if the child had accumulated a minimum of 10 hours of wear time on at least four days of the measurement period. Missing physical activity data were imputed using available values and a combined multiple imputation approach^25^. Additional details of the physical activity measurement procedures have been reported previously^24^.

### Spinal pain outcomes

Spinal pain occurrences were measured using two methods: 1) self-reported pain comprising weekly SMS reports from parents, as well as 2) diagnosed spinal pain identified by clinical examination and audit of linked medical records. Diagnosed spinal pain was further classified as traumatic or non-traumatic.

Measures of self-reported spinal (neck, mid-back or lower back) pain were collected each week using a web-based SMS text messaging system (SMS-Track ApS, Esbjerg, Denmark). This approach is reliable, and valid compared to information from structured clinical interviews^26^. Each Sunday, parents responded to questions about the presence or absence of spinal pain experienced by their child during the previous week, with all responses uploaded to an online database. Parental involvement was utilized owing to concerns over the validity of direct reports from children of this age^27^. Inappropriate responses resulted in a telephone call to parents for clarification.

When reports of spinal pain were received, research staff contacted parents by telephone at the beginning of the subsequent week. If the pain was still present at the time of telephone follow-up, a standardized clinical examination performed by a trained primary health care provider was scheduled within two weeks. If indicated, the child was then referred to an orthopedic surgeon for additional evaluation and paraclinical investigation (e.g., diagnostic imaging). Occurrences of spinal pain were classified using International Classification of Diseases version 10 diagnostic codes (diagnosed spinal pain), with M-codes indicating non-traumatic pain and S-codes indicating spinal pain resulting from trauma^28^. We also collected information on any additional healthcare utilization during the study period through linked medical records. This information was used to inform diagnostic decisions.

### Data analysis

All analyses were performed using STATA 12 (StataCorp, College Station, Texas, USA). Descriptive analyses comprised the proportion (expressed as a percentage) of children who experienced spinal pain, as well as the prevalence of one or more episodes of spinal pain over the course of the study.

Physical activity data were averaged across the two study periods in all inferential analyses. All associations were examined with logistic regression models employing robust standard errors to account for the repeated measures within individuals. We constructed separate logistic regression models to investigate the associations between physical activity exposures and each of the four spinal pain outcomes (self-reported spinal pain, diagnosed spinal pain, traumatic spinal pain, non-traumatic spinal pain). The dichotomous dependent variable was the presence or absence of at least one episode of spinal pain for each outcome. Missing SMS responses were coded as ‘no change’, in reference to the previous week.

Physical activity exposures were measured in two ways. First, we measured overall physical activity with mean counts per minute. To enhance interpretation, these parameter estimates were reported at 100 counts per minute; this means that, relative to the mean counts per minute at baseline, these odds ratios represent the odds of experiencing spinal pain per 18% increase in overall physical activity.

Second, associations with physical activity intensity were examined using the proportion of time spent in sedentary, light, moderate, and vigorous intensity activity. The proportion of time in light, moderate, and vigorous physical activity were simultaneously entered into each model, with the proportion of sedentary time as the reference category. Therefore, given the mean wear time of 13.3 hours/day, these parameter estimates represent the change in odds associated with shifting approximately 8 minutes from sedentary time to another intensity category (e.g., moderate), when time in the other categories (e.g., light and vigorous) remains constant.

Age and sex are risk factors for the development of spinal pain in children^29, 30^. Consequently, we also modeled adjusted odds ratios controlling for sex and using class level as a surrogate for age. The level of significance was 0.05 for all analyses.

### Data availability

Data are available from the CHAMPS Study Steering Committee upon reasonable request. Legal and ethical restrictions apply. Interested parties may contact Dr. Niels Christian Møller (nmoller@health.sdu.dk), and the following information will be required at the time of application: a description of how the data will be used, securely managed, and permanently deleted.

## Results

Overall, 1205 children participated in the study. The mean duration of participation was 36 weeks in period one, 37 weeks in period two and 70 weeks overall. Participant characteristics and spinal pain outcomes across the two study periods are reported in Table 1. The mean weekly response rate to the text messages was 96.5%. There were 1723 (2.3%) weeks of reported spinal pain. Nearly one-third (31%) of children experienced one or more episodes of spinal pain. In total, 14% of the children had diagnosed spinal pain, with 8% classified as non-traumatic and 6% as traumatic. Spinal pain lasting 1–2 weeks represented the most common pain duration, comprising 80% of reported episodes.

**Table 1:** Participant characteristics and spinal pain prevalence stratified by study period and sex^a^.

	Study Period 1^b^	Study Period 2^c^
Sample size	1169	1160
Age, mean ± SD, yr	9.9 ± 1.4	10.4 ± 1.4
Sex, n (% female)	620 (53.0)	612 (52.8)
Weight, mean ± SD, kg	34.6 ± 8.3	36.6 ± 8.9
Height, mean ± SD, cm	141.2 ± 10.1	144.5 ± 10.5
Self-reported spinal pain		
Girls	21.0 (17.8, 24.2)	25.0 (21.6, 28.4)
Boys	18.2 (15.0, 21.5)	19.2 (15.9, 22.5)
Total	19.7 (17.4, 22.0)	22.2 (19.9, 24.6)
Diagnosed spinal pain		
Girls	7.7 (5.6, 9.9)	11.9 (9.4, 14.5)
Boys	6.7 (4.6, 8.8)	5.5 (3.6, 7.4)
Total	7.3 (5.8, 8.8)	8.9 (7.2, 10.5)
Non-traumatic spinal pain		
Girls	4.5 (2.9, 6.2)	7.4 (5.3, 9.4)
Boys	3.5 (1.9, 5.0)	3.3 (1.8, 4.8)
Total	4.0 (2.9, 5.2)	5.4 (4.1, 6.7)
Traumatic spinal pain		
Girls	3.6 (2.1, 5.0)	5.1 (3.3, 6.8)
Boys	3.5 (1.9, 5.0)	2.2 (1.0, 3.4)
Total	3.5 (2.5, 4.6)	3.7 (2.6, 4.8)

^a^Values are percentage (95% CI) unless otherwise indicated. ^b^Mean participation = 36 weeks. ^c^Mean participation = 37 weeks.

Descriptive physical activity statistics are presented in Table 2. Approximately three-quarters (74%) of children accumulated at least 10 hours wear time per day on at least four days of each study period [Mean (SD) valid days = 6.1 (0.9)]. The mean daily wear time was 13.3 hours, and children were sedentary for 62% of the day on average.

**Table 2:** Descriptive accelerometry data^a^ stratified by study period and sex^b^.

	Period 1 (n = 1025)	Period 2 (n = 981)
Wear time per day (hours)		
Girls	13.1 (12.7, 13.6)	13.3 (12.9, 13.8)
Boys	13.3 (12.8, 13.7)	13.4 (12.9, 13.8)
Total	13.2 (12.7, 13.7)	13.4 (12.9, 13.8)
Overall physical activity (CPM)		
Girls	519 (435, 618)	525 (439, 642)
Boys	582 (506, 672)	632 (514, 755)
Total	550 (467, 638)	575 (470, 690)
Percent of day in physical activity intensities		
Sedentary behavior		
Girls	63.0 (58.9, 67.2)	63.5 (58.9, 67.3)
Boys	61.7 (57.9, 65.3)	61.3 (56.6, 64.7)
Total	62.3 (58.3, 66.3)	62.3 (57.8, 66.6)
Light activity		
Girls	29.8 (26.3, 32.7)	29.4 (26.2, 33.0)
Boys	29.5 (26.8, 32.6)	29.5 (26.3, 32.8)
Total	29.6 (26.5, 32.6)	29.5 (26.3, 32.9)
Moderate activity		
Girls	4.7 (3.8, 5.6)	4.6 (3.6, 5.3)
Boys	5.8 (4.8, 6.6)	5.8 (4.7, 7.0)
Total	5.2 (4.2, 6.2)	5.0 (4.1, 6.1)
Vigorous activity		
Girls	2.3 (1.7, 3.1)	2.4 (1.7, 3.3)
Boys	3.0 (2.2, 3.9)	3.4 (2.4, 4.7)
Total	2.6 (1.9, 3.5)	2.9 (1.9, 4.0)

^a^Each physical activity assessment comprised measures obtained over seven consecutive days. ^b^Values are median (interquartile range). CPM = counts per minute.

### Associations between physical activity intensity and spinal pain outcomes

There were no crude associations between overall mean physical activity intensity and any of the spinal pain outcomes. After adjusting for age and sex, increasing mean physical activity intensity by 18% (100 CPM) was associated with increased diagnosed [OR (95%CI) = 1.15 (1.05 to 1.25)] and traumatic [OR (95%CI) = 1.14 (1.00 to 1.28)] spinal pain (Table 3).

**Table 3:** Crude and adjusted associations of mean overall physical activity (100 counts per minute) with spinal pain outcomes^a^.

Self-reported spinal pain	Diagnosed spinal pain	Non-traumatic spinal pain	Traumatic spinal pain
0.99 (0.92, 1.06)	1.06 (0.96, 1.15)	1.02 (0.89, 1.15)	1.08 (0.95, 1.21)
1.05 (0.97, 1.13)^b^	**1.15 (1.05, 1.25)** ^b^	1.13 (1.00, 1.26)^b^	**1.14 (1.00, 1.28)** ^b^

^a^Values are odds ratios (95% confidence intervals) and represent the odds of experiencing spinal pain per 18% increase in counts per minute. Bolded values are statistically significant.

^b^Adjusted for age and sex.

**Figure 1:**
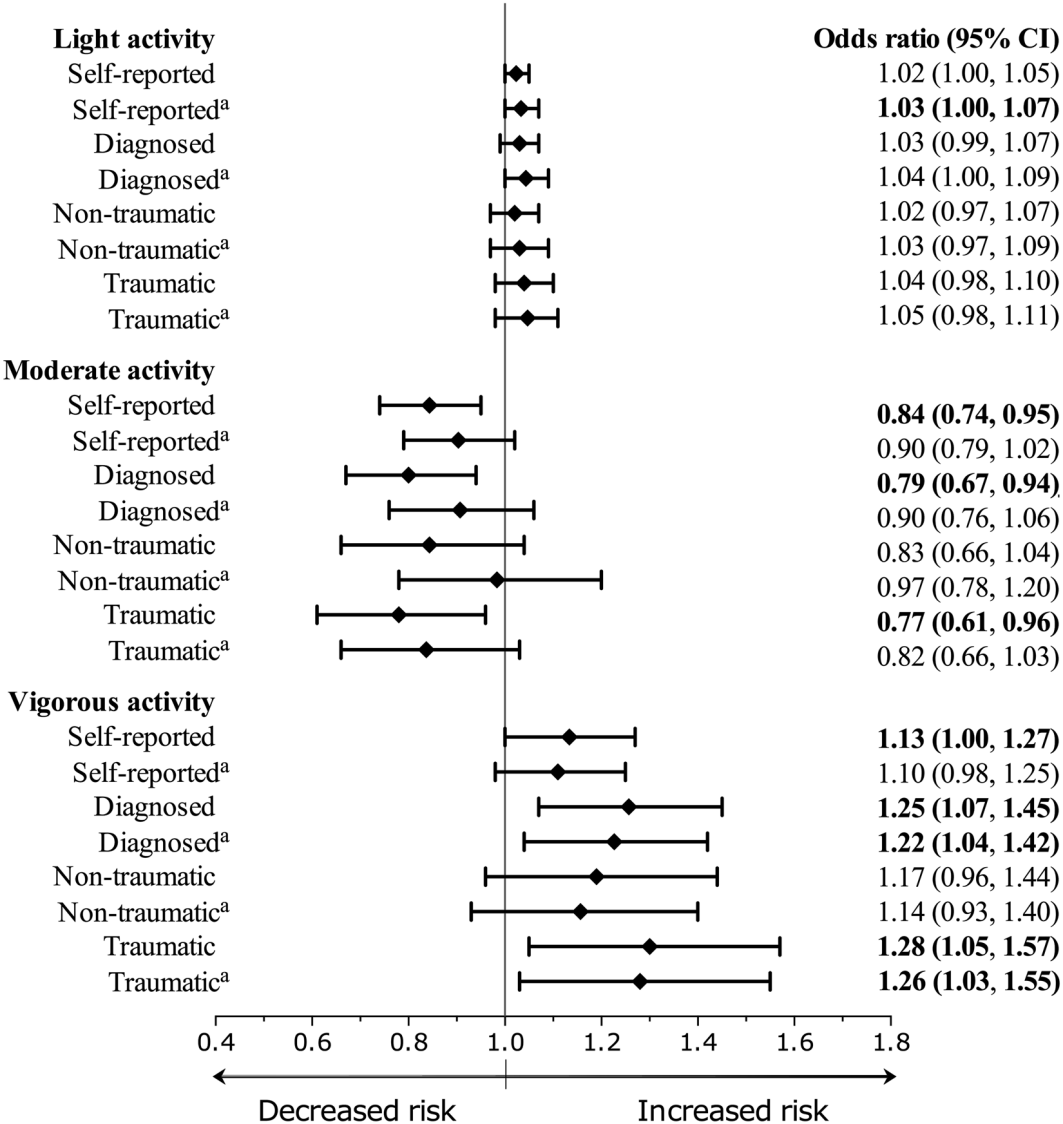
Crude and adjusted odds ratios demonstrating the associations between physical activity intensities and self-reported, diagnosed, non-traumatic, and traumatic spinal pain. Values represent the odds of spinal pain per 8-minute reduction in sedentary time. ^a^Adjusted for age and sex.

Moderate intensity physical activity was associated with reduced odds of self-reported spinal pain [OR (95%CI) = 0.84 (0.74 to 0.95)], diagnosed spinal pain [OR (95%CI) = 0.79 (0.67 to 0.94)] and traumatic spinal pain [OR (95%CI) = 0.77 (0.61 to 0.96)]. Vigorous intensity physical activity was associated with increased self-reported [OR (95%CI) = 1.13 (1.00 to 1.27)], diagnosed [OR (95%CI) = 1.25 (1.07 to 1.45)] and traumatic [OR (95%CI) = 1.28 (1.05 to 1.57)] spinal pain (Fig. 1). After controlling for age and sex, light physical activity intensity physical activity was associated with increased self-reported spinal pain [OR (95%CI) = 1.03 (1.00 to 1.07)], while vigorous intensity physical activity was associated with increased diagnosed [OR (95%CI) = 1.22 (1.04 to 1.42)] and traumatic [OR (95%CI) = 1.26 (1.03 to 1.55)] spinal pain (Fig. 1).

## Discussion

We found physical activity behavior to be associated with future spinal pain in children. Children who engaged in higher levels of overall physical activity were more likely to experience some types of spinal pain; however, the nature of this relationship depended on the intensity of physical activity. Shifting from time spent in sedentary activities to vigorous physical activities was associated with increased occurrences of spinal pain. Conversely, shifting from sedentary to moderate intensity activities tended to protect against spinal pain, while increased time in light intensity activity had no consistent association with spinal pain. Traumatic spinal pain demonstrated the largest associations with time in moderate (protective) and vigorous (increased risk) intensity physical activities. These results suggest that physical activity intensity is a key consideration when seeking to understand the role of physical activity behavior in the development of spinal pain in children.

Our findings differ with previous longitudinal studies investigating the relationships between objectively measured physical activity behavior and self-reported spinal pain. This may be explained by differences in the measurement of physical activity exposures or spinal pain outcomes.

Aartun *et al*.^15^, found no associations between overall physical activity levels or different activity intensities and spinal pain in young people aged 11 to 15 years. A secondary (subgroup) analysis of this study isolated an inception cohort of 144 young people who, at baseline, reported no history of previous spinal pain^31^. Time spent in sedentary, moderate-to-vigorous, or vigorous physical activities did not predict future spinal pain. However, participants in the 90^th^ percentile of time in vigorous physical activity had an increased risk of spinal pain (relative risk [95% CI] = 1.26 [1.00 to 1.58] to 1.44 [1.09 to 1.91]).

There are two noteworthy differences with the current study. First, several analyses in that study combined moderate and vigorous activity intensities into a single physical activity category. Our study results suggest that moderate and vigorous physical activity have opposing effects on the development of spinal pain. Therefore, combining intensity categories may 'wash out' the potential﻿﻿﻿ effects of moderate and vigorous intensity physical activity. The second difference involves the measures of spinal pain. Aartun *et al*.^15, 31^, asked participants to self-report their lifetime history of spinal pain, while we prospectively measured spinal pain each week over the course of the study. Moreover, we went beyond self-reported pain by including information from physical and paraclinical examinations and classified each occurrence as traumatic or non-traumatic. The latter approach helped to exclude transient pain episodes, which may explain the stronger associations observed with diagnosed, versus self-reported, spinal pain. Therefore, our measure of ‘diagnosed’ spinal pain may represent a more clinically relevant assessment.

Our group has investigated the prospective associations of physical activity behavior of children and adolescents with self-reported spinal pain in two previous studies^16, 17^. While we reported no consistent associations between overall physical activity and spinal pain^16, 17^, high-intensity physical activity protected against future spinal pain^17^. Differences in the physical activity measures, including the use of different accelerometers and cut-points of physical activity intensity, make direct comparisons difficult. Additionally, the one-minute epoch length (compared to 10 seconds in the current study), likely led to differential categorization of physical activity intensities. As accelerometry measurement protocols evolve, the true nature of physical activity behavior will continue to emerge. Therefore, we consider the current study findings to have advanced knowledge on the relationships between physical activity behavior and spinal pain.

The major strengths of this study are the longitudinal design, large representative sample, and methods of measuring both physical activity exposures and spinal pain outcomes. Physical activity was objectively measured, with standard approaches that are more accurate than subjective estimates^32^. The participating children had a high level of adherence to the accelerometry protocol. Spinal pain outcomes were prospectively measured using intensive data collection methods. By including information from clinical and paraclinical examinations, we advanced traditional approaches that solely rely on self-reporting. The high weekly adherence rates of 96.5% support the validity of the spinal pain data and demonstrate the feasibility of collecting such information with intensive monitoring.

While preferable to self-reported physical activity, accelerometry has several inherent limitations that represent potentials source of residual confounding in our analyses. Accelerometry is unable to capture all modes of physical activity (e.g., cycling, swimming). There are no widely accepted thresholds of activity intensity cut-points. Consequently, we were unable to measure all forms of physical activity, and it is possible that some activity was misclassified. Additionally, the use of accelerometry requires the assumption that the physical activity outcomes represent the individual’s usual behaviour. Despite using accelerometry best practices, it is possible that some measures were not representative of each child’s usual level of physical activity. In study period one, the measures of physical activity preceded all spinal pain measures, but it is possible that some reports of spinal pain occurred prior to the second physical activity measure. However, the low occurrence of new spinal pain cases and averaging of data between periods would have diminished the potential for temporal bias arising from this limitation. We accounted for two potential sources of confounding (class level and sex); however, the relatively small number of cases and need to account for clustering effects prevented us from exploring for additional covariates. Moreover, previous longitudinal studies have failed to identify consistent risk factors of back pain in young people^33^, making informed analysis decisions regarding the inclusion of additional covariates challenging.

Physical activity behavior and spinal pain represent two important public health problems. It is, therefore, important to have a clear understanding of these results from a public health perspective. The World Health Organization recommends that children engage in at least 60 minutes of moderate-to-vigorous physical activity per day to improve health and prevent non-communicable diseases^34^. Our results demonstrate that moderate intensity physical activity may protect against the development of spinal pain in children and support current recommendations for health-related physical activity. Our results also suggest that vigorous intensity physical activity may be a modifiable risk factor for the development of future spinal pain in children. It is important to note that the current study results should not lead to recommendations for children to avoid vigorous physical activities. These results represent a first step toward understanding the relationship between vigorous physical activity and musculoskeletal disorders, such as spinal pain, from a risk-benefit perspective.

It will be the role of future research to further our understanding of the relations between physical activity behavior and spinal pain in youth and adulthood. Advancing knowledge of the role of vigorous physical activity in the development of spinal pain, and the potential protective effects of moderate intensity physical activity, will require additional large-scale prospective studies and randomized clinical trials. Our findings are consistent with a previous study that identified a U-shaped distribution between self-reported physical activity intensity and back pain in adults^35^, and this may represent a useful perspective for future research investigating this relationship. Achieving consensus on optimal accelerometry practices will improve the validity of physical activity measures as well as comparability across studies, including data synthesis. Future studies should emphasize clinically relevant estimates of spinal pain. Finally, understanding the physical activity and pain trajectories experienced by young people will enhance our understanding of these important public health problems.

## Conclusion

We found physical activity behavior to be associated with future spinal pain in children. While greater overall physical activity was associated with increased occurrences of spinal pain, the nature of this relationship depended on the intensity of activity. Increased time in vigorous physical activity predicted future spinal pain, while increased time in moderate intensity activities tended to protect against spinal pain. These results suggest that physical activity intensity is a key consideration when seeking to understand the role of physical activity behavior in the development of spinal pain in children.
